# Supplementary material for: Mechanistic basis for PYROXD1-mediated protection of the human tRNA ligase complex against oxidative inactivation
Source: Nat Struct Mol Biol. 2025 Mar 11;32(7):1205–12. doi: 10.1038/s41594-025-01516-6 (PMC12263435; doi:10.1038/s41594-025-01516-6)

Fig. 2C

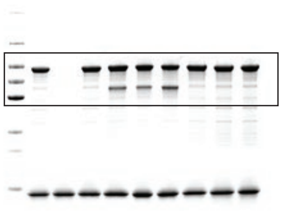

Fig. 2D

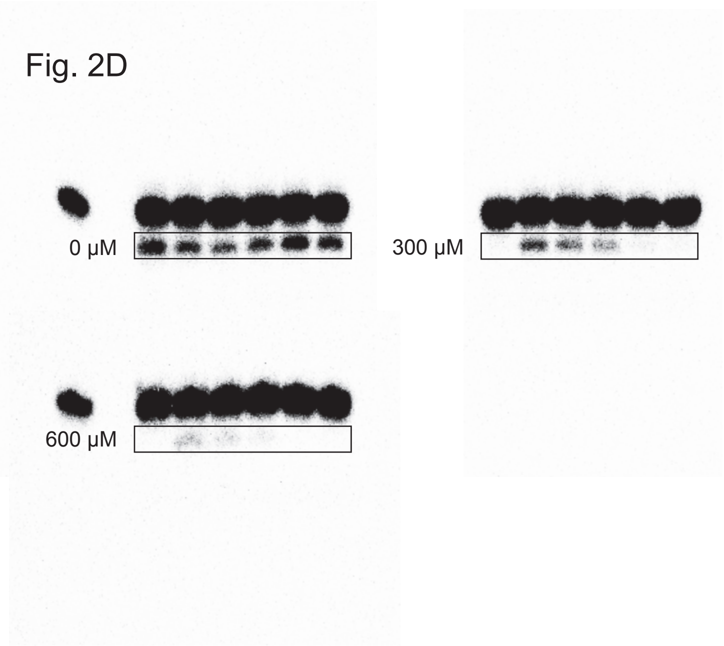

Fig. 3D

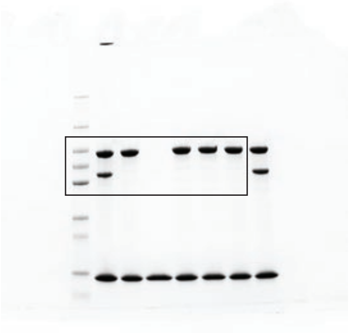

Fig. 3E

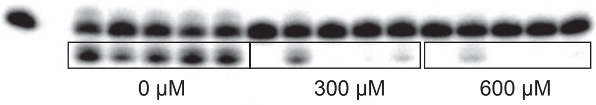

Fig. 4B + Extended Data Fig. 4H

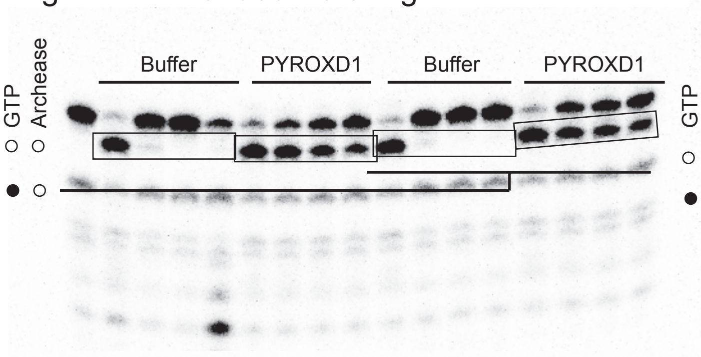

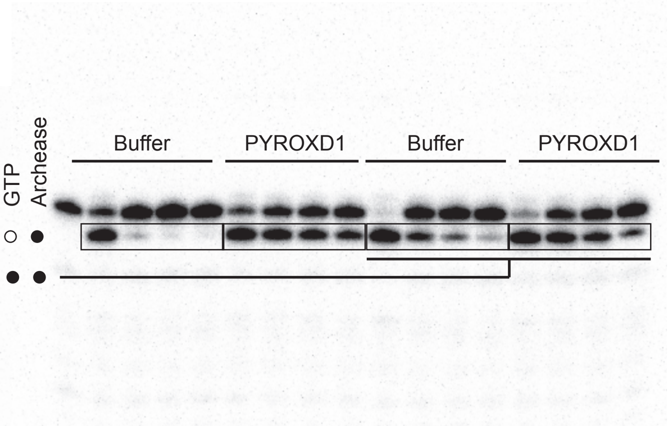

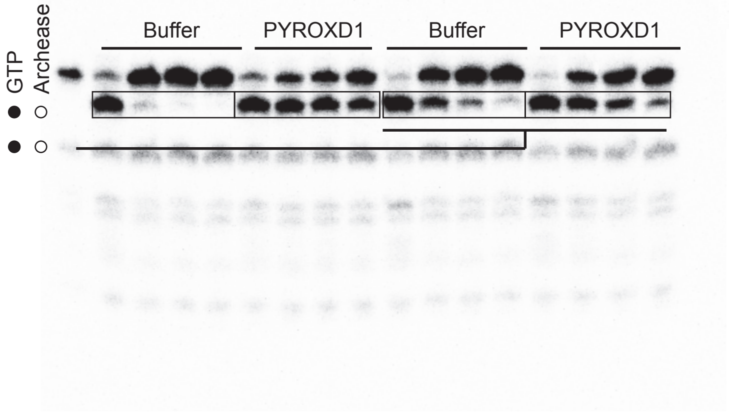

Fig. 4C

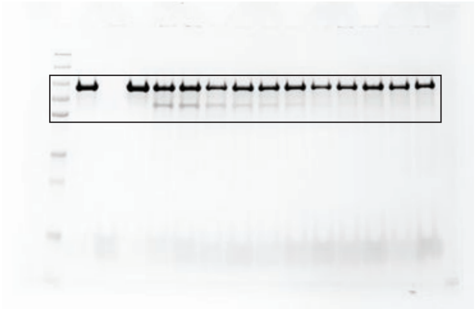

Extended Data Fig. 1A

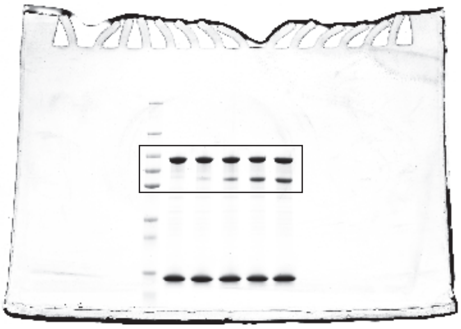

Extended Data Fig. 1B

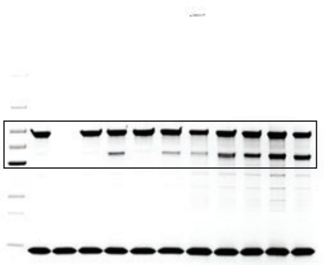

Extended Data Fig. 4I

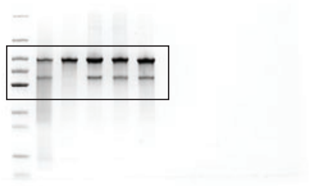

Supplement: Supplementary file 4 — Uncropped gels. [file 41594_2025_1516_MOESM4_ESM.pdf]
